# Crossroads: advanced guidance through an aortic coarctation by fusion imaging in transfemoral TAVR after aorto-aortic bypass

**DOI:** 10.1007/s12928-021-00772-9

**Published:** 2021-03-29

**Authors:** Martin Geyer, Alexander R. Tamm, Felix Kreidel, Andres Beiras-Fernandez, Thomas Münzel, Ralph Stephan von Bardeleben

**Affiliations:** 1 Department of Cardiology, Cardiology I, University Medical Center of the Johannes Gutenberg-University Mainz, Langenbeckstr. 1, 55131 Mainz, Germany; 2 Department of Cardiothoracic and Vascular Surgery, University Medical Center of the Johannes Gutenberg-University, Langenbeckstr. 1, 55131 Mainz, Germany

**Keywords:** Aortic stenosis, TAVI, Aortic coarctation, Fusion imaging, Multidisciplinary heart team

A 78-year-old male patient with symptomatic aortic valve stenosis and history of surgical therapy of a preductal aortic coarctation by an aorto-aortic bypass was admitted to our Heart Center (Fig. 1; A: 3D-reconstruction from CT/B: fluoroscopy/C: fusion imaging; elongated native aortic arch (a) with coarctation (arrow), and aorto-aortic bypass (b)). Echocardiography and CT scan showed a degenerated and severely calcified bicuspid aortic valve (Type I LR, according to Sievers classification [1], D). Severe annular calcification was favoring the selection of a self-expandable prosthesis. Intricacy of vascular access for transcatheter aortic valve replacement (TAVR) was relevantly increased due to the patient’s special anatomy with an intact aortic bypass graft bearing a large appositional thrombus (**, E), whereas the patent native aortic arch was stenotic and elongated in kind of a “double-z” shape with two stenotic segments (smallest vessel diameter 9 mm in a 135° curve directly distal to the ostium of the left carotid artery, see arrow in A and red color markers in C). As an alternative subclavian access was unfavorable due to angulation and vessel diameter, we decided for the “long way” in kind of transfemoral approach via the native kinked aortic arch.

**Fig. 1:**
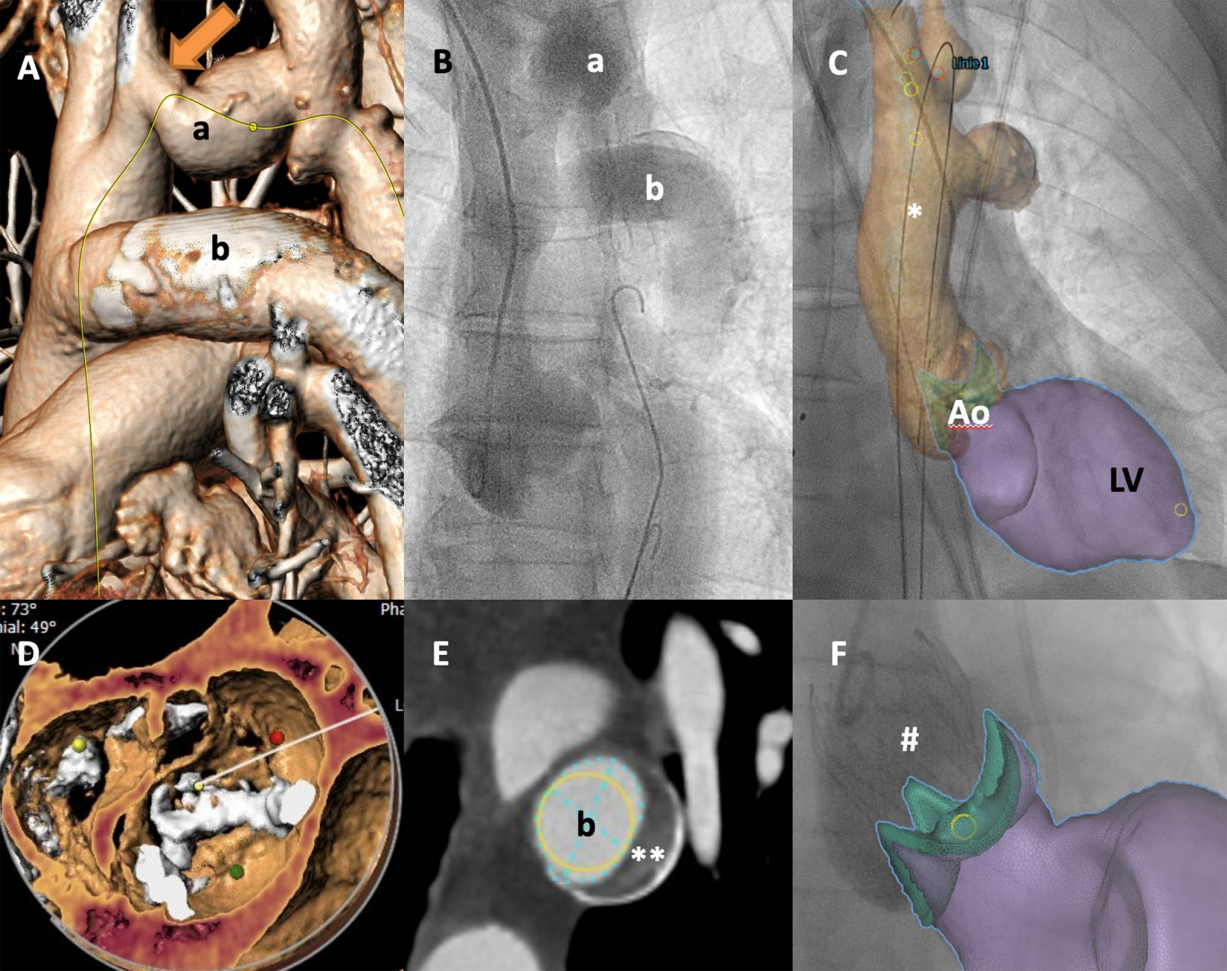
**A** CT-reconstruction of “double” aortic arch: elongated, stenotic native aortic arch (a) and aorto-aortic bypass (b), arrow indicating smallest vessel diameter of the coarctation. **B** Fluoroscopy (via radial pigtail-catheter, tip in non-coronary sinus): native aortic arch (a) and aorto-aortic bypass (b). **C** Fusion imaging (LV, left ventricle; Ao, aortic valve), stiff wire (*) guided by real-time fusion imaging through native stenotic aortic arch (color markers indicating stenotic segment). **D** CT-reconstruction of native aortic valve stenosis (n. b. calcified fusion of left and right coronary cusp/bicuspid aortic valve Typ I LR). **E** CT-reconstruction of aorto-aortic bypass (b) with appositional thrombus (**). **F** Fusion imaging: result aortography showing self-expanding TAVR-prosthesis (#) in situ

Implementation of fusion imaging of both fluoroscopy and the screening CT scan entailed safe guidance of the stiff wire (asterisk, C; shape of the coarctation’s angulation only moderately affected by the guidewire) through the aortic coarctation (see also supplemental video). A Medtronic EvolutPro® 29 mm (Medtronic, Minneapolis, MN, USA) TAVR-prosthesis (#, F) was implanted without any problems after pre-dilatation by balloon-valvuloplasty (peak-to-peak gradient > 100 mmHg) with a good result of excellent hemodynamics and mild paravalvular leakage.

Our case demonstrates that advanced guidance by fusion imaging can facilitate safe TAVR-procedures even in uncommon and complicated anatomies.

## Supplementary Information

Below is the link to the electronic supplementary material.

Fusion imaging: fusion of real-time fluoroscopy and planning CT-scan: the TAVR-prosthesis is advanced over the stiff wire (tip left ventricle via native aortic valve, as depicted by colored CT-overlay) through the native kinked and stenotic aortic arch (the smallest vessel diameter part of the stenosis is marked by red circles and blue line). For details, see text. (MP4 12627 kb)
